# Immune Checkpoint Inhibitors-Associated Myocarditis: Diagnosis, Treatment and Current Status on Rechallenge

**DOI:** 10.3390/jcm12247737

**Published:** 2023-12-17

**Authors:** Federica Frascaro, Nicola Bianchi, Federico Sanguettoli, Federico Marchini, Sofia Meossi, Luca Zanarelli, Elisabetta Tonet, Matteo Serenelli, Gabriele Guardigli, Gianluca Campo, Luana Calabrò, Rita Pavasini

**Affiliations:** 1 UO Cardiologia, Azienda Ospedaliero-Universitaria di Ferrara, 44124 Ferrara, Italy; federica.frascaro@edu.unife.it (F.F.); bncncl1@unife.it (N.B.); federico.sanguettoli@edu.unife.it (F.S.); mrcfrc2@unife.it (F.M.); sofia.meossi@unife.it (S.M.); luca.zanarelli@edu.unife.it (L.Z.); tonet.elisabetta@gmail.com (E.T.); m.serenelli@ospfe.it (M.S.); gabriele.guardigli@unife.it (G.G.); cmpglc@unife.it (G.C.); 2 Dipartimento di Medicina Translazionale e per la Romagna, Univerity of Ferrara, 44121 Ferrara, Italy; luana.calabro@unife.it; 3 UO Medical Oncology, Azienda Ospedaliero-Universitaria di Ferrara, 44124 Ferrara, Italy

**Keywords:** immune checkpoint inhibitors related myocarditis, cardio oncology, rechallenge

## Abstract

Immune checkpoint molecules like cytotoxic T-lymphocyte antigen 4 (CTLA-4), programmed cell death 1 (PD-1) or its ligand, programmed cell death ligand 1 (PD-L1), play a critical role in regulating the immune response, and immune checkpoint inhibitors (ICIs) targeting these checkpoints have shown clinical efficacy in cancer treatment; however, their use is associated with immune-related adverse events (irAEs), including cardiac complications. The prevalence of cardiac irAEs, particularly myocarditis, is relatively low, but they can become a severe and potentially life-threatening condition, usually occurring shortly after initiating ICI treatment; moreover, diagnosing ICI-related myocarditis can be challenging. Diagnostic tools include serum cardiac biomarkers, electrocardiography (ECG), echocardiography, cardiac magnetic resonance (CMR) and endomyocardial biopsy (EMB). The treatment of ICI-induced myocarditis involves high-dose corticosteroids, which have been shown to reduce the risk of major adverse cardiac events (MACE). In refractory cases, second-line immunosuppressive drugs may be considered, although their effectiveness is based on limited data. The mortality rates of ICI-induced myocarditis, particularly in severe cases, are high (38–46%). Therapy rechallenge after myocarditis is associated with a risk of recurrence and severe complications. The decision to rechallenge should be made on a case-by-case basis, involving a multidisciplinary team of cardiologists and oncologists. Further research and guidance are needed to optimize the management of cancer patients who have experienced such complications, evaluating the risks and benefits of therapy rechallenge. The purpose of this review is to summarize the available evidence on cardiovascular complications from ICI therapy, with a particular focus on myocarditis and, specifically, the rechallenge of immunotherapy after a cardiac adverse event.

## 1. Introduction

Immune checkpoints, like cytotoxic T-lymphocyte antigen 4 (CTLA-4), programmed cell death 1 (PD-1) or its ligand, programmed cell death ligand 1 (PD-L1), are negative immunoregulatory molecules that inhibit excessive T cell activation in the body. A tumor microenvironment includes a plethora of immune cells that recognize cancer neoantigens and, once activated, proliferate to eliminate malignant cells [1]. However, cancer cells can up-regulate the PD-L1 expression on their surface to escape from immune system recognition. Immune checkpoint inhibitors (ICI), blocking immune checkpoints at different levels, remove the inhibitory brake and make cancer cells visible again to the immune system [1]. ICIs approved by the Food and Drug Administration and from the European Medicines Agencies (EMA) include an inhibitor of CTLA-4 (ipilimumab), inhibitors of PD-1 (nivolumab, pembrolizumab and cemiplimab) and inhibitors of PD-L1 (atezolizumab, avelumab and durvalumab) [1]. The benefit of ICIs has expanded in several settings of malignancies thanks to their clinical efficacy in improving overall survival with reduced adverse events. Unfortunately, the augmentation of systemic autoreactive T cells can lead to various immune-related adverse events (irAEs) in different tissues, such as the skin, thyroid, intestines, nervous system and heart [1,2,3]. The organs most frequently affected by adverse events after ICI depend on the type of drug administered as follows: for example, in the case of ipilimumab administration, adverse events are dermatological, gastrointestinal and renal; for pembrolizumab arthralgia, pneumonitis and hepatic toxicities, for nivolumab, endocrine toxicities, and for atezolizumab, hypothyroidism [4]. Cardiac and neurological adverse events are rarer [4] but more severe, especially myocarditis, and, in particular, the fulminant one has very high mortality rates [5]. The pathophysiology of irAEs is not well understood; several hypotheses have been put forward considering the factors that together contribute to altering immunological homeostasis, such as the loss of self-tolerance, the cross-reactivity between cancer antigens and normal tissue ones, and the increasing levels of preexisting autoantibodies and inflammatory cytokines [2].

The aim of this narrative review is to summarize all the available evidence about cardiovascular complications of ICIs therapy, particularly myocarditis. Moreover, the possibility of ICI rechallenge after cardiovascular irAEs will be analyzed, as this remains a very controversial aspect of ICI treatment with little evidence.

### Methodological Consideration

We performed an extensive literature research using various databases, including PubMed, Web of Science and Scopus, using the MeSH strategy and looking for the following terms: ((immune-check point inhibitors) OR (ICI)) AND ((myocarditis) OR (cardiovascular event) OR (cardiac adverse event) OR (myocarditis) OR (troponin) OR (heart failure)). Only manuscripts published in English and in peer-reviewed journals were selected. We also checked for the references of relevant reviews on this topic to be more inclusive. We included manuscripts regarding the occurrence of a cardiac adverse event (defined as myocarditis, cardiogenic shock, arrhythmic events, heart failure, increase in troponin level during treatment, pericardial disease or other adverse events possibly related to cardiac involvement) after ICI treatment. No restriction regarding the study type was performed. A literature search and screening of the literature was performed by four independent reviewers (NB, FF, FS, FM). Divergences were solved by discussion and consensus. In the case of unresolved disagreement, another reviewer (RP) tried to reach a consensus. Four reviewers (NB, FF, FS, FM) retrieved data from the included studies. Next, we applied a systematic methodology to the research of case reports on the rechallenge of ICI therapy (details in Section 3.6).

## 2. ICI Induced Cardiac Adverse Events

Considering data from a recent metanalysis including 4751 patients, the prevalence of cardiac irAEs can be estimated around 1.3% and it is higher for patients receiving dual ICI therapy [6]. The main cardiac disorders related to irAEs post-ICI therapy are myocarditis, arrythmias, heart failure and pericardial effusion [6]. ICI-related myocarditis can cause various arrhythmias from atrial fibrillation, supraventricular arrhythmias to ventricular tachycardias and atrioventricular (AV) blocks. Atrial tachyarrhythmias are also reported in ICI-related thyrotoxicosis [6]. At the same time, ICIs have been associated with a 3-fold higher risk of atherosclerotic cardiovascular events due to plaque progression. The pathophysiological mechanism underlying the acceleration of atherosclerotic plaque formation and destabilization during ICI therapy is not fully understood, but is likely related to the inflammation and immune dysregulation (ICI therapies, in particular, modulate T cell activation finally promoting atherosclerosis) [7]. As such, it is important to stratify patients with a higher pre-therapy cardiovascular risk who may be at an increased risk of immuno-related plaque destabilization seen that this phenomenon can be attenuated with concomitant use of statins and corticosteroids [8]. Instead, pericardial diseases can manifest with pericarditis, pericardial effusion or even cardiac tamponade. Current data about prevalence of pericardial disease post-ICI are limited. Findings derived from case reports and series shows that a pericardial disease after ICI therapy occurs early and especially in males [1]. Other non-inflammatory cardiac irAEs manifestations include Takotsubo cardiomyopathy, vasculitis, non-inflammatory heart failure and left ventricular disfunction [9,10].

## 3. ICI-Associated Myocarditis

Myocarditis is the most frequent and feared complication of ICIs therapy and its incidence varies between 0.27% and 1.14% (with a probable underestimation due to asymptomatic cases) with a mortality rate of up to 50% [1,2,3]. Generally, it occurs early with a median time of from 17 to 34 days from ICIs initiation and after from 1 to 2 doses of immunotherapy. An ICI combination is the stronger established risk-factor for developing myocarditis.

The mechanistic basis of immune checkpoint inhibitor (ICI)-associated myocarditis remains unclear. While T cell-mediated immunity is implicated, several questions persist, including the identification of cardiac antigens, the reasons for immune response elicitation, the roles of innate immune and B cells, the involvement of specific cardiac cell types, manifestations of cardiac cellular dysfunction and the significance of cell death programs [11]. Genetic studies in mice highlight the importance of T cells and immune checkpoints, such as CTLA-4 and PD-1, in myocarditis pathogenesis. Environmental factors impact autoimmune myocarditis, and genetic backgrounds influence the outcomes of disruptions in the PD-1 axis. Notably, the coexistence of CTLA-4 and PD-1 disruptions in mice mimics a combined anti-CTLA-4/anti-PD-1 therapy, a risk factor for ICI-associated myocarditis, with CTLA-4-Ig intervention attenuating disease progression [11]. Questions arise about the antigens triggering T cell responses in ICI-associated myocarditis and why these antigens evade tolerance mechanisms. While cardiac proteins like myosin and β-adrenergic receptors are implicated, the specific antigens and mechanisms remain uncertain [11]. The nature of heart injury and the extent of direct immune attack on cardiomyocytes versus damage through other cell types warrants further investigation. Understanding how the immune system damages the heart, whether through cell death, dysfunction, or both, and identifying the involved cell types and regulatory mechanisms are critical areas for future research [11].

The estimation of the risk of cardiotoxicity before starting ICI therapy is complex and one must take into account multiple parameters, such as age, sex, cardiovascular risk factors (smoking, dyslipidemia, hypertension, family history of cardiovascular diseases), previous cardiac disease or previous cardiotoxicity from chemotherapy [12] and the Heart Failure Association-International Cardio-Oncology Society (HFA-ICOS) risk score can be used as a useful tool for risk stratification.

Myocarditis resulting from dual ICI therapy is also more likely to be severe and fatal (65.6% versus 44.4% in dual ICI versus monotherapy) [13].

Other risk factors include concomitant cardiotoxic agents (e.g., anthracycline) or underlying autoimmune diseases (e.g., lupus, sarcoidosis, rheumatoid arthritis) [1].

### 3.1. Clinical Presentation of ICI-Associated Myocarditis

The clinical presentation of myocarditis associated with ICI can vary from asymptomatic elevation in cardiac biomarkers to end-organ failure. Symptoms and presentation can be challenging ranging from chest pain, dyspnea, but also myalgia, myasthenia, ptosis, muscle weakness, syncope, palpitation, pulmonary edema and cardiogenic shock. Myocarditis can begin with conduction delay, atrial fibrillation and ventricular arrhythmias, which can lead to sudden death [1,9].

A diagnosis must be suspected in the presence of symptoms, laboratory and electrocardiographic (ECG) changes suggestive of myocardial damage. However, no parameter is pathognomonic of ICIs-related myocarditis, so other causes of myocardial injury like acute coronary syndrome (ACS) should be excluded. Other, more specific differential diagnoses, are the myocardial involvement in drug reaction with eosinophilia and systemic symptoms (DRESS) syndrome [14] or during cytokine release syndrome during ICI therapy (defined as reactions seen after the administration of targeted therapies that cause hyper-activation of T cells that recognize tumor antigen [15]). 

DRESS is a severe adverse drug reaction with a delayed onset (median latency 40 days, less in lethal cases) after drug administration, and unlike ICI-associated myocarditis, its manifestation includes fever and eosinophilia. More common cardiac symptoms of DRESS are dyspnea, cardiogenic shock, chest pain, tachycardia, cardiac arrest or pericardial effusion. It has a mortality rate up to 10% (even more, up to 45% in patients with cardiac involvement) and also causes the development of a progressive renal impairment. It often relapses once steroid therapy is tapered. Due its rare, various presentations, DRESS is largely underdiagnosed and its pathogenesis in relation to ICI is uncertain [14,16].

If patients present with other irAEs, the diagnostic suspicion is stronger; in 25% of cases, myocarditis is associated with myositis, and in 10% of cases with myasthenia gravis [1].

During and after ICI therapy, it is important to monitor for the onset of symptoms that may be associated with cardiovascular side effects, like myocarditis or the effect of accelerated atherosclerosis. Patients should also be educated about how to recognize these symptoms. Additionally, it is useful to implement a structured cardiovascular screening program for early identification of cardiovascular complications (primarily myocarditis) according to the protocols suggested by the latest European Society of Cardiology (ESC) guidelines [12], which include the following: (i) electrocardiogram (ECG), troponin and brain natriuretic peptide (BNP) measurements before starting therapy; (ii) echocardiogram before starting therapy (mandatory in high-risk patients); (iii) repeating ECG and troponin measurements after the second, third and fourth doses of ICI, and then every three doses thereafter; (iv) comprehensive cardiology evaluation every 6–12 months in patients requiring long-term ICI therapy (mandatory in high-risk patients).

No preventive pharmacologic therapy is known to be effective in reducing the risk of ICI-related cardiotoxicity, but in patients with previous cardiovascular disease, it is useful to optimize cardiac-specific medical therapy and in patients with high cardiovascular risk to optimize risk factors control [12].

### 3.2. Diagnostic Testing for ICI-Associated Myocarditis

**Figure 1:**
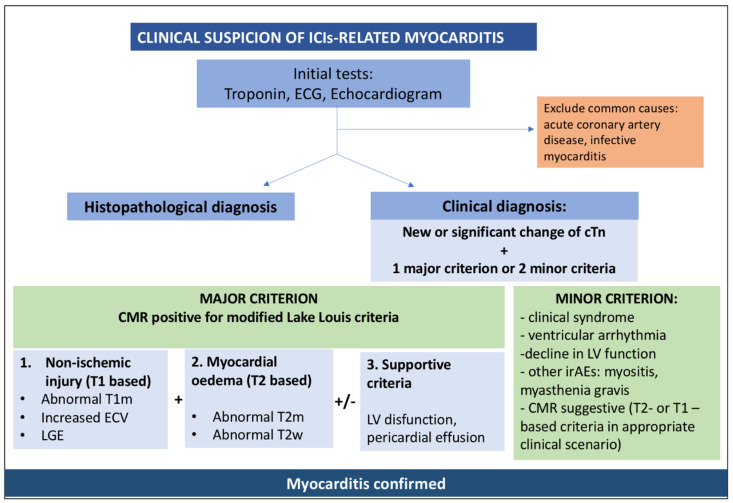
The role of cardiac imaging for the diagnosis of immune checkpoint inhibitor-induced myocarditis. cTn, cardiac troponin; CMR, cardiac magnetic resonance; ECG, electrocardiogram; ECV, extracellular volume; LGE, late gadolinium enhancement; irAEs, immune-related adverse events; LV disfunction, left ventricular disfunction; T1m, T1 mapping; T1w, T1 weighted; T2m, T2 mapping; T2w, T2 weighted.

The presentation of patients with ICI-associated myocarditis is generally associated to elevation of serum cardiac biomarkers, such as cardiac troponin and creatine kinase-muscle/brain (CK-MB) (Figure 1).

Since troponin T may be increased during myositis, troponin I assessment is recommended in suspicion of ICIs-myocarditis. N-terminal prohormone of brain natriuretic peptide (NT-proBNP) can be elevated, but its specificity is lower and can be persistently elevated in many cancer patients [17].

A troponin assay assumes not only a diagnostic but also a prognostic value because a persistent elevation of serum biomarkers have been associated with negative outcomes [1,18]. In a multicentric registry, patients that experienced major adverse cardiac events (MACE) defined as composite of cardiovascular death, cardiac arrest, cardiogenic shock and hemodynamically significant complete heart block had a troponin T value at admission and discharge higher than others; in particular, at discharge, the troponin T value was ≥1.5 ng/mL [17].

Data confirming the prognostic role of cardiac biomarkers also come from the presence of some case reports of ICI-related myocarditis in which mortality also remains high in asymptomatic cases with only positive biomarkers (although lower than in symptomatic forms) and it is around 16% [18].

ECG may show characteristic changes like prolongation of a PR interval, atrioventricular blocks, ventricular arrhythmias, ST depression and diffuse negative T waves.

Echocardiography might be normal or show reduced ventricular function with regional wall motion abnormalities in the acute phase, whereas dilatation and remodeling suggest a chronic manifestation [18]. A normal left ventricular ejection fraction (LVEF) does not rule out myocarditis because cardiac function is normal in about 51% of patients and 38% of those who develop MACEs (defined as composite of cardiovascular death, cardiac arrest, cardiogenic shock and hemodynamically significant complete heart block) have normal LVEF [18]. As stated before, the current ESC guidelines suggest that all patients on ICI therapy should have an ECG and troponin assay at baseline, whereas high-risk patients should have, in addition, an echocardiographic evaluation [12].

The global longitudinal strain (GLS) is a sensitive parameter used to monitor the left ventricular function in cancer patients treated with traditional cytotoxic agents. The European guidelines recommended to determinate GLS in all cancer patients and to start cardioprotective drugs if GLS decreases by at least 15% in patients undergoing treatment with anthracyclines or HER2-target therapy [12]. However, there are no specific indications for patients treated with ICIs. Several studies demonstrated the utility of GLS to predict subclinical cardiac toxicities also in patients treated with ICIs [19,20]. A recent retrospective analysis compared GLS values between patients who developed ICI-myocarditis and those without adverse events, and demonstrated that GLS was reduced only among patients who developed myocarditis; furthermore, GLS was reduced either in patients presenting with a reduced LVEF and in those with a preserved LVEF. In addition, the decline in GLS was found to predict MACE (defined as composite of cardiovascular death, cardiac arrest, cardiogenic shock and hemodynamically significant complete heart block) regardless of LVEF [21].

Endomyocardial biopsy (EMB) is considered the gold standard diagnostic test in case of suspicion of ICI-related myocarditis; according to the Dallas criteria, myocarditis is diagnosed in the setting of an “inflammatory infiltrate of the myocardium with necrosis and/or degeneration of adjacent myocytes, not typical of ischemic damage associated with coronary artery disease” [22,23].

**Figure 2:**
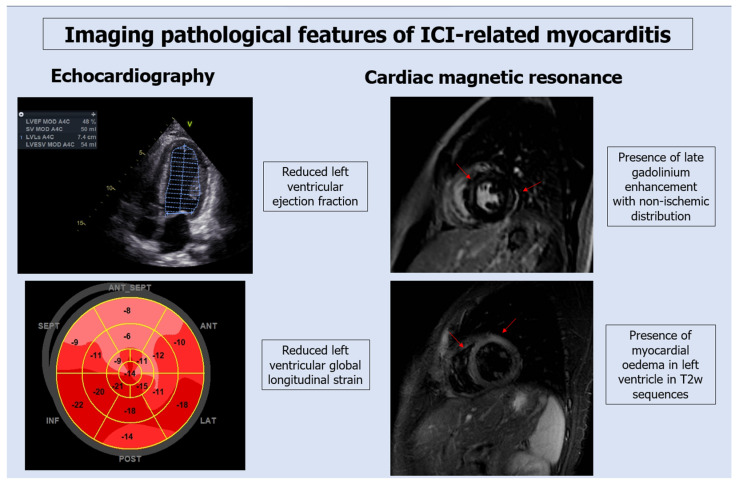
Echocardiographic and magnetic resonance imaging in a patient with ICI-related myocarditis. Echocardiography shows an impaired left ventricular ejection fraction with a reduction in global longitudinal strain with a non-coronary distribution. Magnetic resonance shows presence of late gadolinium enhancement (red arrows in top right figure) in inversion recovery gradient echo (IRGE) sequences and myocardial edema (red arrows in bottom right figure) in T2-weighted sequences.

The ICOS published a new diagnostic definition of ICI-myocarditis based on the elevation of troponin associated with a major criterion or 2 minor criteria. Cardiac magnetic resonance (CMR) remains the stronger diagnostic tool to identify edema and scar formation and it is listed as a major criterion [24]. Although the modified 2018 Lake Louis (2018-LL) criteria have not been validated for the diagnosis of ICI-myocarditis, their use is recommended by guidelines [12]. They are based on the presence at CMR of myocardial edema (global or regional increase in myocardial native T2 relaxation time or T2 signal intensity) and non-ischemic myocardial injury (identified by a global or regional increase in myocardial native T1 mapping or extracellular volume fraction [ECV] or regional late gadolinium enhancement [LGE] signal increase) [22] (Figure 2). A recent study demonstrated that the LL criteria have low sensitivity for the diagnosis of ICI-myocarditis because these patients have a lower rate of LGE and, when present, it is more frequently localized in the midseptum. Moreover, septal LGE in these patients proved to be the only CMR predictor of MACE (defined as death from cardiovascular causes (including sudden death), documented sustained (>30 s) ventricular tachycardia, ventricular fibrillation, complete atrioventricular heart block and cardiogenic shock) at 1 year, independently of troponin values [25]. In this setting, the presence of only one of the two LL criteria can be considered a suggestive CMR for ICI myocarditis and it is classified by current guidelines as a minor criterion [12] (Figure 1).

When CMR is contraindicated, cardiac fluorodeoxyglucose positron emission tomography (PET) can be used to assess the presence of myocardial inflammation [1,12].

Among other minor criteria, the following are listed: (i) clinical syndrome (fatigue, myalgia, chest pain, diplopia, ptosis, shortness of breath, orthopnea, lower extremity oedema, palpitations, dizziness, syncope, muscle weakness, cardiogenic shock); (ii) ventricular arrythmias or new conductance disturbance; (iii) decline in LV function; (iv) other immune-related adverse events (myositis, myasthenia, myopathy) [12].

Given the diagnostic difficulty of ICI-related myocarditis, research should also focus on new diagnostic modalities, such as finding new serum biomarkers that are more specific for this type of myocarditis. Like, for example, the measurement of certain miRNAs has been proposed for the differential diagnosis between acute myocardial infarction and myocarditis [26].

All patients should be classified according to the severity of the disease in fulminant myocarditis (hemodynamically or electrically instable patients), non-fulminant and steroid refractory myocarditis (worsening state despite corticosteroids) [22].

The America Society of Clinical Oncology (ASCO) clinical practice guidelines divide myocarditis into the following four degrees of severity: grade 1 (asymptomatic patients with abnormal ECG or troponin), grade 2 (poorly symptomatic patients with altered troponin and ECG), grade 3 (moderate symptomatic patients with diagnostic or suggestive echocardiogram or CMR), grade 4 (life threatening disease with cardiac study abnormalities in grade 1–3).

### 3.3. Treatment

In the suspicion of myocarditis, it is recommended to hold ICI, even for a mild grade of toxicity [23,27].

If ICI-myocarditis is suspected, cardiotoxic therapy should be discontinued regardless of the severity of myocarditis, and the patient should be hospitalized with continuous ECG monitoring [12].

First-line therapy is based on the early initiation of high-dose corticosteroids since patients who receive cortisone early (less than 24 h) and at higher doses (500–1000 mg per day) have a lower risk of MACE (defined as cardiovascular death, cardiac arrest, cardiogenic shock and hemodynamically significant complete heart block requiring pacemaker) and lower discharged troponins [3]. Cardio-oncology guidelines recommend starting therapy with methylprednisolone 500–1000 mg i.v. bolus for 3–5 days and, only in case of clinical response (symptom resolution, troponin reduction of at least 50%, no evidence of arrhythmias or AV blocks), it is recommended to switch to oral prednisolone 1 mg/kg [12,28,29].

The tapering of prednisolone should be performed very slowly, about 10 mg per week under troponin and cardiac function monitoring [12]. If the troponin value starts to increase as soon as the steroid dosage is reduced, it is necessary to increase the dosage again and taper over a longer period [23].

In the case of steroid refractory (persistently increased troponin, no improvement in cardiac function, electrical instability), it is necessary to switch to second-line immunosuppressive drugs, like mycophenolate mofetil, anti-thymocyte globulin (anti-CD3 antibody), i.v. immunoglobulin, plasma exchange, tocilizumab, abatacept (CTLA-4 agonist), alemtuzumab (anti-CD52 antibody) and tofacitinib. Unfortunately, no randomized studies have analyzed the effectiveness of these therapeutic strategies and clinical information derived from case reports [29].

Caution should be used before using infliximab (antitumor necrosis factor-alpha (TNF-α)) because it has been shown to deteriorate conditions in patients with acute heart failure [29,30].

**Figure 3:**
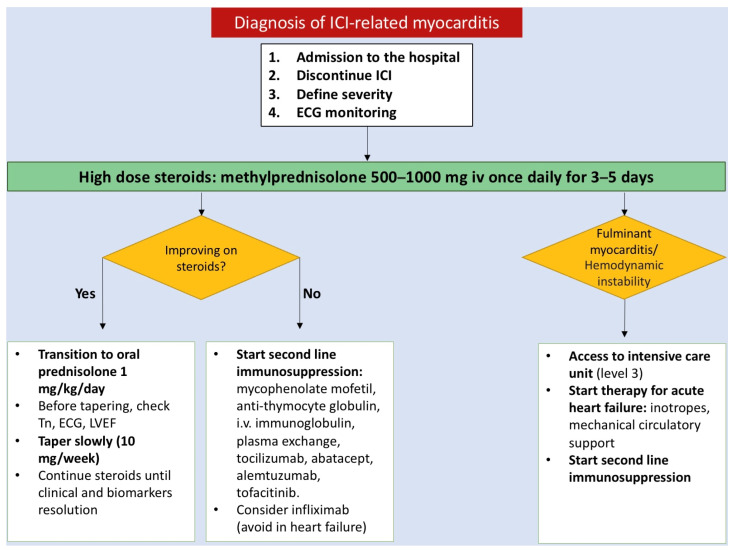
Clinical management of immune checkpoint inhibitor-induced myocarditis. ECG, electrocardiogram; ICI; immune checkpoint inhibitor; iv, intravenous; LVEF, left ventricular ejection fraction; Tn, troponin.

Patients with hemodynamic instability should be admitted to the intensive care unit and they should be managed following the acute and chronic heart failure guidelines; for acute heart failure, intravenous diuretics, inotropes and circulatory supports are recommended [12] (Figure 3).

### 3.4. Outcomes

ICI-myocarditis demands close attention due to its significant impact on patient outcomes. In fact, fatal events were reported in from 38% to 46% of patients, even when appropriate first- and second-line treatments are administered [16,31,32].

To address this critical concern, the ASCO and ESC guidelines recommends cardiological screening of patients before starting ICI therapy (including ECG and troponin). Underdiagnosis of subclinical forms of myocarditis might be present. The mortality rate previously reported primarily reflected clinically evident and severe cases of ICI-myocarditis with possible overestimation of mortality [33,34].

Beyond the immediate risks posed by ICI-myocarditis, the use of ICI drugs in cancer therapy has also raised concerns about long-term cardiovascular complications derived from inflammation and immune dysregulation. Research has indicated an increase in cardiovascular risk associated with treatment with these drugs, as well as a threefold rise in the progression of total aortic plaque volume (rate of progression of aortic plaque volume on CT scan from 2.1%/year to 6.7%/year) [8]. However, it is important to acknowledge that evidence supporting this association between ICIs and long-term cardiovascular is limited, and in some cases, data came from animal models. Considering all of this, all patients treated with ICIs, especially those who experienced cardiac adverse events, should benefit from long-term cardiovascular follow-up.

### 3.5. Relapse and Therapy Rechallenge

Given the severity of myocarditis as a potential adverse event, the ASCO guidelines do not endorse the reinitiation of therapy after grade 1 toxicity, which encompasses abnormal cardiac biomarker test results or ECG abnormalities [27].

Consequently, there is a paucity of data regarding the relapse of ICI-myocarditis after rechallenging patients with ICI drugs. This caution surrounding rechallenge can be seen as a prudent preventive strategy, considering the findings of a study conducted by Simonaggio et al. [35].

Simonaggio et al. examined 93 patients who experienced immunological non-cardiac toxicity (the most common were hepatitis, cutaneous adverse events, pneumonitis, colitis and arthritis) of grade ≥ 2, and of those, 43 patients were rechallenged with ICI drugs. The results were that 55% of these individuals experienced immunological adverse events following rechallenge, with 42.5% of cases involving a recurrence of the same type of toxicity, even if never more severe than the first [35]. Given the life-threatening nature of myocarditis, these results can be concerning if extrapolated into this scenario and underscores the need for a comprehensive risk-benefit analysis before considering rechallenge.

Rechallenging with ICI therapy in the presence of a history of myocarditis should be approached with the utmost caution. Decisions regarding rechallenge should be made on a patient-by-patient basis, involving a multidisciplinary team that includes both cardiologists and oncologists [36]. If the decision to rechallenge is made, it is recommended to implement a rigorous monitoring strategy, which includes serial assessments of biomarkers and echocardiography [37].

Despite the growing awareness of this issue, the body of the literature addressing the rechallenge of ICI therapy after myocarditis remains limited. Only a handful of case reports and series exists that shed light on the practical aspects and challenges of this delicate decision-making process.

### 3.6. Review of Case Reports on Rechallenge after ICI Myocarditis

With the aim to evaluate the state of the art about the rechallenge after ICI myocarditis, we performed a specific literature review on PubMed, Scopus and Web of Science using the following terms using MeSh strategy: ((immunocheck point inhibitors) OR (ICI)) AND rechallenge AND ((myocarditis) OR (cardiovascular event) OR (cardiac adverse event) OR (myocarditis) OR (troponin) OR (heart failure)). As already detailed, only papers published in English and in peer-review journals were selected. Only i) observational or randomized clinical trials and ii) case reports were included regarding the use of rechallenge after a cardiac adverse event related to ICI therapy. No randomized clinical trials or observational trials focused on rechallenge were found. A literature search and screening of the literature were performed by two independent reviewers (SM, LZ). Divergences were solved by discussion and consensus. In the case of unresolved disagreement, another reviewer (GC) tried to reach a consensus. Two reviewers (ET, MS) retrieved data from the included studies. Only 9 case reports were focused on this topic, including 16 patients overall (Table 1).

As observed, the rechallenge of treatment was considered in all grades of previous myocarditis. This strategy, despite its potential benefits, was not without risks. In four cases, the rechallenge strategy was ineffective due to cardiac-related issues, such as a recurrence of myocarditis or a worsening of cardiac symptoms. Notably, these challenges were not encountered in patients who had initially been diagnosed with grade 4 myocarditis, suggesting that myocarditis severity is not the only element involved in relapse risk.

On the other hand, in two unfortunate cases, patients, despite undergoing therapy rechallenge, ultimately succumbed to cancer progression in less than a year. These cases bring to the forefront the following two important considerations: the need for meticulous assessment of the risk-benefit balance when contemplating therapy rechallenge. The oncologist plays a central role in this process, tasked with evaluating the potential benefits of rechallenge and determining whether they outweigh the potentially life-threatening risk of myocarditis recurrence. Secondly, the paucity of data on this extremely difficult clinical scenario highlights the need for clinical studies focused on this item.

## 4. Conclusions

In conclusion, this narrative review emphasizes the significance of cardiovascular adverse events associated with immune checkpoint inhibitors (ICIs) in cancer therapy. While ICIs have revolutionized the field of oncology by improving overall survival, they come with the risk of irAEs, including myocarditis and other cardiac complications.

Myocarditis, the most frequent and potentially life-threatening cardiac complication of ICIs, manifests in various forms, often leading to severe outcomes such as cardiogenic shock, cardiac arrest and arrhythmias. The prevalence of cardiac irAEs is relatively low but can be more common in patients receiving combination ICI therapy. Diagnosing ICI-related myocarditis can be challenging, as no single parameter is pathognomonic. Finally, there is a gap in the existing literature, particularly about the rechallenge of therapy after ICI-myocarditis. Given the complex risk-benefit balance involved in such decisions, further research and guidance are needed to optimize the management of cancer patients who have experienced cardiovascular complications related to ICIs.

## Figures and Tables

**Table 1:** Case reports and series of rechallenge of ICI after myocarditis.

Reference	Number of Patients Rechallenged	Grade of Myocarditis			Adverse Event after Rechallenging
		Grade 1	Grade 2–3	Grade 4	
Xue Chen et al., 2022 [38]	5	0	5	0	1 patient: grade 2 myocarditis 1 patient: cancer death after 1 year
Peleg Hasson S. et al., 2021 [39]	3	1	2	0	1 patient: worsening cardiac symptoms
Dae Hyun Lee et al., 2020 [40]	1	0	0	1	None
Menachery Sherin M. et al., 2023 [41]	1	0	0	1	Cancer death after <1 year
Eslinger Cody et al., 2023 [42]	1	0	0	1	None
Rossi A. Valentina et al., 2023 [43]	1	0	1	0	Grade 1 myocarditis
Yeshan Chen et al., 2022 [44]	1	0	1	0	None
Dinu Valentin Balanescu et al., 2020 [45]	2	0	2	0	None
Shen et. al., 2021 [46]	1	0	1	0	Grade 2 myocarditis

## Data Availability

No new data were created or analyzed in this study. Data sharing is not applicable to this article.

## Abbreviations

CMR, cardiac magnetic resonance; ECG, electrocardiogram; EMB, endomyocardial biopsy; ETT, transthoracic echocardiogram; HF, heart failure; ICU, intensive care unit; i.v., intravenous; mLL criteria, modified Lake Louis criteria; NT-proBNP, N-terminal prohormone of brain natriuretic peptide.
